# Patient and System-Related Delays of Emergency Medical Services Use in Acute ST-Elevation Myocardial Infarction: Results from the Third Gulf Registry of Acute Coronary Events (Gulf RACE-3Ps)

**DOI:** 10.1371/journal.pone.0147385

**Published:** 2016-01-25

**Authors:** Khalid F. AlHabib, Kadhim Sulaiman, Jassim Al Suwaidi, Wael Almahmeed, Alawi A. Alsheikh-Ali, Haitham Amin, Mohammed Al Jarallah, Hussam F. Alfaleh, Prashanth Panduranga, Ahmad Hersi, Tarek Kashour, Zohair Al Aseri, Anhar Ullah, Hani B. Altaradi, Kazi Nur Asfina, Robert C. Welsh, Salim Yusuf

**Affiliations:** 1 Department of Cardiac Sciences, King Fahad Cardiac Center, College of Medicine, King Saud University, Riyadh, Kingdom of Saudi Arabia; 2 Department of Cardiology, Royal Hospital, Muscat, Oman; 3 Department of Cardiology, Hamad Medical Corporation (HMC), Doha, Qatar; 4 Heart and Vascular Institute, Cleveland Clinic, Abu Dhabi, United Arab Emirates; 5 College of Medicine, Mohammed Bin Rashid University of Medicine and Health Sciences, Dubai, United Arab Emirates; 6 Institute of Cardiac Sciences, Sheikh Khalifa Medical City, Abu Dhabi, United Arab Emirates; 7 Tufts Clinical and Translational Science Institute, Tufts Medical Center, Boston, MA, United States of America; 8 Mohammed Bin Khalifa Cardiac Center, Manama, Bahrain; 9 Sabah Al-Ahmed Cardiac Center, Kuwait, Kuwait; 10 Emergency Department, College of Medicine, King Saud University, Riyadh, Kingdom of Saudi Arabia; 11 Division of Cardiology, Mazankowski Alberta Heart Institute, University of Alberta, Edmonton, Canada; 12 Population Health Research Institute, Hamilton Health Sciences, McMaster University, Hamilton, Canada; University of Bologna, ITALY

## Abstract

**Background:**

Little is known about Emergency Medical Services (EMS) use and pre-hospital triage of patients with acute ST-elevation myocardial infarction (STEMI) in Arabian Gulf countries.

**Methods:**

Clinical arrival and acute care within 24 h of STEMI symptom onset were compared between patients transferred by EMS (Red Crescent and Inter-Hospital) and those transferred by non-EMS means. Data were retrieved from a prospective registry of 36 hospitals in 6 Arabian Gulf countries, from January 2014 to January 2015.

**Results:**

We enrolled 2,928 patients; mean age, 52.7 (SD ±11.8) years; 90% men; and 61.7% non-Arabian Gulf citizens. Only 753 patients (25.7%) used EMS; which was mostly via Inter-Hospital EMS (22%) rather than direct transfer from the scene to the hospital by the Red Crescent (3.7%). Compared to the non-EMS group, the EMS group was more likely to arrive initially at a primary or secondary health care facility; thus, they had longer median symptom-onset-to-emergency department arrival times (218 vs. 158 min; *p*˂.001); they were more likely to receive primary percutaneous coronary interventions (62% vs. 40.5%, *p* = 0.02); they had shorter door-to-needle times (38 vs. 42 min; *p* = .04); and shorter door-to-balloon times (47 vs. 83 min; *p*˂.001). High EMS use was independently predicted mostly by primary/secondary school educational levels and low or moderate socioeconomic status. Low EMS use was predicted by a history of angina and history of percutaneous coronary intervention. The groups had similar in-hospital deaths and outcomes.

**Conclusion:**

Most acute STEMI patients in the Arabian Gulf region did not use EMS services. Improving Red Crescent infrastructure, establishing integrated STEMI networks, and launching educational public campaigns are top health care system priorities.

## Introduction

Acute reperfusion therapy is critical for improving the outcomes of patients with ST-elevation myocardial infarction (STEMI). The guidelines have advocated that timely primary percutaneous coronary intervention (PPCI) in experienced centers is a superior mode of therapy compared to thrombolytic therapy (TT) [1,2]. Quality improvement initiatives, such as the “Stent for Life” in Europe, the “D2B Alliance” and the “Mission:Lifeline” in the USA, were established to improve the rate of timely PPCI for patients with STEMI [3–7]. However, adopting a pharmaco-invasive approach has also been considered a reasonable therapeutic strategy in cases where PPCI could not be achieved within 90–120 min from first medical contact. The pharmaco-invasive approach involves treatment with anti-platelets and TT (pre- or in-hospital), followed immediately by a rescue PCI, when reperfusion fails, or by an elective PCI within 6–24 h, when reperfusion is successful [8–10]. Integrated, well-organized STEMI networks facilitate choosing the optimal acute reperfusion strategy according to the individual clinical scenario [11–16].

Emergency Medical Services (EMS) is a major cornerstone in the acute STEMI chain of survival. EMS provides prompt recognition and early management of acute STEMI, which are critical in reducing morbidity and mortality [1,2]. We demonstrated previously that only one in four patients with STEMI used the EMS system in the Arabian Gulf region; also, short and long-term outcomes were similar to patients that used other means of transport (non-EMS). However, in that study, the majority of patients that used EMS were treated with in-hospital TT rather than PPCI [17]. Accordingly, we intended to develop STEMI networks across our region with a major emphasis on increasing the rate of timely PPCI. To that end, we acknowledged that an intimate understanding of the EMS triaging system for patients with acute STEMI in our communities was required to establish an effective STEMI system of care. Thus, we undertook the third phase of the project termed the *Gulf R*egistry of *A*cute *C*oronary *E*vents: *P*rimary *P*CI *P*rograms (Gulf RACE-3Ps), with the following objectives: (1) Assess temporal changes in clinical arrivals and EMS usage rates compared to our previous reports; (2) describe the EMS system (Red Crescent and Inter-Hospital); (3) compare the clinical presentation, management, and outcomes in the emergency department (ED) and in the hospital between patients that used EMS (EMS group) and those that used other means of transport (non-EMS group), and between hospitals that provided PCI (PCI-hospitals) and those that did not provide PCI (non-PCI hospitals).

## Materials and Methods

### Study Population

The Gulf RACE-3Ps is a prospective, multicenter study that has recruited consecutive patients with acute STEMI that were treated in hospitals in Saudi Arabia, Oman, United Arab Emirates, Kuwait, Qatar, and Bahrain. A pilot phase was conducted from May to December 2013 to assess feasibility; those data are not included in the present report. The present study population included patients with acute STEMI, aged ≥18 years, which showed clinical indications for acute revascularization therapies within 24 h of symptom onset. Because we were interested mainly in the acute management of patients with STEMI, we excluded patients that were transferred for an elective coronary angiogram after 24 h of symptom onset. Unlike the well-developed EMS systems in western countries, the EMS in our region is quite dispersed, and it is under the authority of two main health care providers: (1) Red Crescent EMS: ambulances operated by the Red Crescent transfer patients directly from the scene to the nearest hospital (PCI or non-PCI); (2) Inter-Hospital EMS: ambulances under the authority of non-PCI hospitals that transfer patients to the nearest PCI hospital, or from a peripheral and small-sized non-PCI hospital to a larger non-PCI hospital that is equipped with an intensive care unit, then subsequent transfer to a PCI hospital whenever possible.

### Study Objectives and Definitions

#### 1. EMS care of patients with acute STEMI

The objective was to describe the sequence of events and the management processes, starting from the onset of symptoms and ending when the patient arrived at the hospital. This included: the proportion of patients with acute STEMI transported to the hospital by EMS (EMS group), and whether the ambulances were under the authority of the Red Crescent. This also included data on the infrastructure of the ambulance services: the level of training and services offered by ambulance personnel; i.e., basic life support (BLS), advanced cardiac life support (ACLS), physicians on-board, and performance of pre-hospital ECG; and the EMS response time. We used standard definitions of acute STEMI and acute revascularization therapies, based on the American College of Cardiology Foundation/American Heart Association and the European Society of Cardiology guidelines [1,2]. General patient demographics, clinical presentation, and in-hospital management variables were defined according to the standard definitions used in the previous phase of the registry (Gulf RACE-2) [18]. Additionally, we collected data on patient socioeconomic status (SES), arbitrarily defined by the average household monthly income, as follows: low SES <$1000/month; moderate SES, $1000-5000/month; and high SES ˃$5000/month. Education was classified as illiterate, primary school, secondary school, Diploma, University, Masters, and PhD. Ethnicities included Arabs, South Asians, and “other”, which refers to all other ethnicities combined into a single group. First medical contact was defined as the first point of the patient’s contact with a clinic, ED, or a doctor (by phone). The total ischemic time was measured from symptom onset to reperfusion therapy with either TT or PPCI.

#### 2. Emergency department and in-hospital care of patients with acute STEMI: EMS vs. non-EMS care

The objective was to study the relationships between the mode of transportation (EMS versus non-EMS) and processes of care for patients with acute STEMI in the ED (door-to-ECG time, and door-to-needle time”DNT”) and in the hospital (door-to-balloon time, “DBT”). We compared the use of evidence-based treatments within the first 24 h of hospital admission, left ventricular assessments with echocardiography, coronary revascularization procedures, in-hospital outcomes, and mortality rates. We also stratified the EMS and non-EMS groups further, according to whether they were transferred by the Red Crescent EMS versus Inter-Hospital EMS versus non-EMS, and whether they were initially managed at a PCI-hospital or non-PCI hospital.

### Study Organization

Information for each patient with STEMI was completed upon hospital admission and at the end of the hospital stay by assigned physicians and/or research assistants working in each hospital. The data were verified by a cardiologist, then sent electronically to the principal coordinating center, where they were further checked. Ethics approval was obtained from the institutional review board (IRB) of King Khalid University Hospital, King Saud University, Riyadh, Saudi Arabia, as well as from all participating hospitals. The list of these hospitals was published previously in the Gulf RACE-2 study [18]. Given that the study is in part a quality improvement initiative, and that patients identity were anonymized to the analyzers, the IRB did not require a written informed consent.

## Statistical Analysis

We evaluated the medical history, clinical presentation, socio-demographic variables, anthropometric measurements, in-hospital course, treatments, and patient outcomes, including mortality. These data were compared between the EMS and non-EMS groups. Categorical data were summarized as frequency and percentages; continuous data were summarized as the mean and SD or median and IQR, based on whether the distribution satisfied the normality assumption. We used a Chi-square test or Fisher’s exact test to compare categorical variables, as appropriate. Between-group comparisons of quantitative variables were carried out with independent *t*-tests or the Mann-Whitney U test, based on the normality assumption. Multiple logistic regression models, with stepwise selection and backward elimination, were used to identify predictors of EMS use. The predictors included in the full model were age, gender, nationality, ethnicity, education, monthly income, type of STEMI, diabetes, hypertension, history of MI, history of angina, and history of PCI. We reported summary statistics and *p*-values. All Statistical analyses were performed with SAS version 9.2 (SAS Institute, Inc, Cary, NC). All data were doubled-checked for errors prior to analyses. We considered a *p* <0.05 to indicate statistical significance.

## Results

### Overall STEMI Cohort

**Fig 1:**
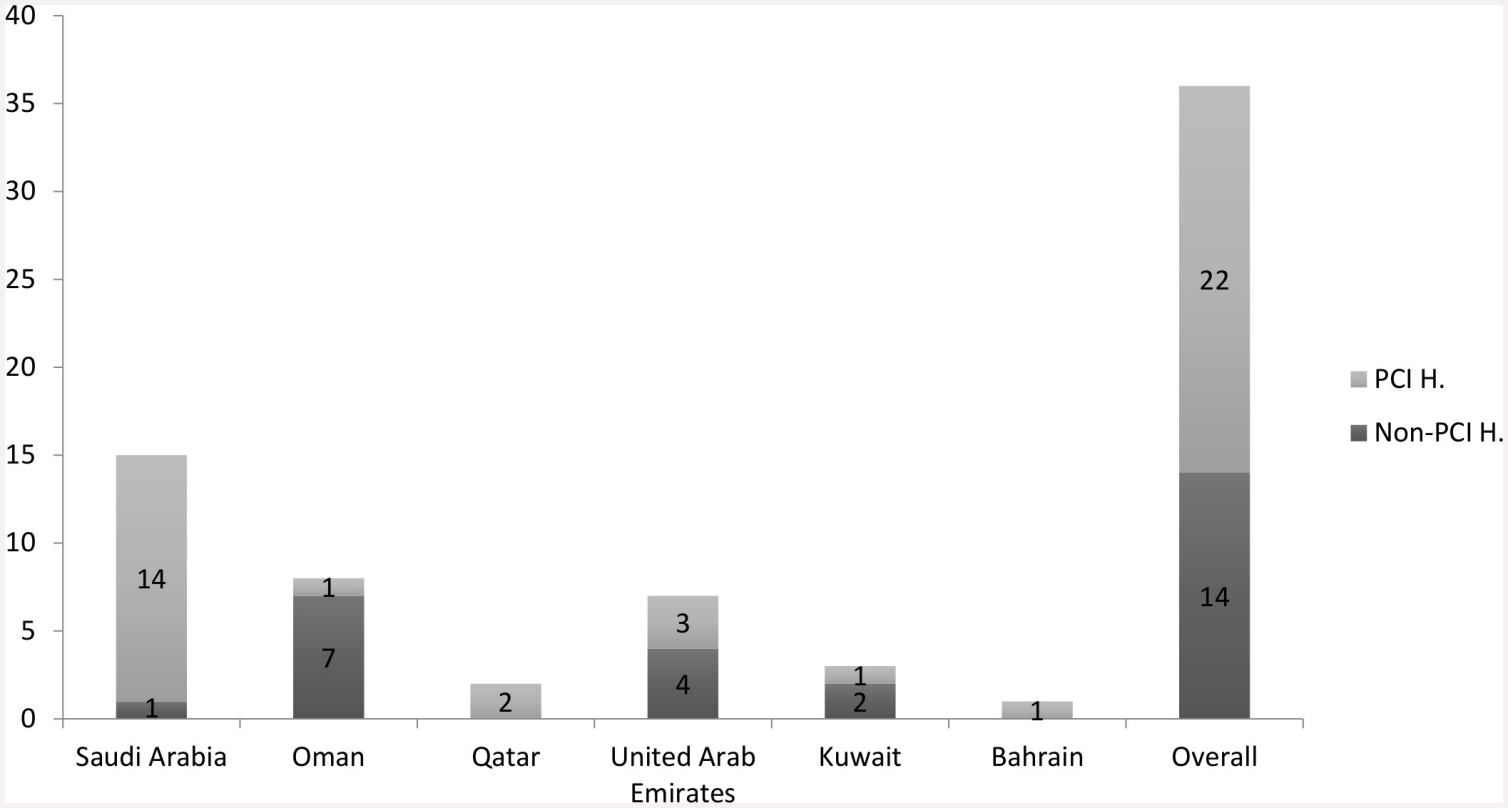
Number of hospitals with percutaneous coronary intervention capability (PCI H.) versus without (Non-PCI H.) that enrolled acute STEMI patients in the study per each Arabian Gulf country.

From 1 January 2014 to 15 January 2015, we enrolled 2,928 patients with acute STEMI from 36 hospitals in 6 Arabian Gulf countries. Among the hospitals, 22 were PCI hospitals, and most of these were available to perform PPCI 24 h per day, 7 days per week (24/7); the other 14 were non-PCI hospitals (Fig 1). The cohort included mostly males (90%), non-Gulf citizens (61.7%), and the mean age (± SD) was 52.7 ± 11.8 years. Acute anterior STEMI had occurred in 53.7% (Table 1).

**Table 1:** Demographics, clinical features, education, and socioeconomic characteristics of patients with acute STEMI transported to the hospital by an emergency medical service (EMS) or alternative transportation (non-EMS).

	Total n = 2928	EMS n = 753 (25.7%)	Non-EMS n = 2175 (74.3%)	*p*-value
	**Row Percentages**			
**Country, n (%)**				
**Oman**	684 (23.4)	133 (19.4)	551 (80.6)	< .001
**Kuwait**	241 (8.2)	21 (8.7)	220 (91.3)	
**Qatar**	476 (16.3)	245 (51.5)	231 (48.5)	
**United Arab Emirates**	299 (10.2)	32 (10.7)	267 (89.3)	
**Bahrain**	140 (4.9)	57 (40.7)	83 (59.3)	
**Saudi Arabia**	1088 (37.2)	265 (24.4)	823 (75.6)	
**Citizenship, n (%)**				
**Gulf**	1123 (38.4)	287 (25.6)	836 (74.4)	0.87
**Non-Gulf**	1805 (61.6)	466 (25.8)	1339 (74.2)	
**Ethnicity, n (%)**				
**Arab**	1123 (38.4)	287 (25.6)	836 (74.4)	0.01
**South Asian**	1353 (46.2)	325 (24)	1028 (76)	
**Other**	452 (15.4)	141 (31.2)	311 (68.8)	
**Type of STEMI, n (%)**				
**Anterior**	1573 (53.7)	415 (26.4)	1158 (73.6)	0.24
**Inferior**	1163 (39.7)	282 (24.3)	881 (75.7)	
**Other**	192 (6.6)	56 (29.2)	136 (70.8)	
**Education, n (%)**				
**Illiterate**	717 (24.5)	148 (20.6)	569 (79.4)	< .001
**Primary School/Secondary School**	1596 (54.5)	413 (26)	1183 (74.1)	
**Diploma/University/Master/PhD**	615 (21)	192 (31.2)	423 (68.8)	
**Average Household Monthly Income, n (%)**				
**< $1000**	1655 (56.5)	407 (24.6)	1248 (75.4)	0.02
**$1000–$5000**	1032 (35.3)	294 (28.5)	738 (71.5)	
**> $5000**	241 (8.2)	52 (21.6)	189 (78.4)	
	**Column Percentages**			
**Age, Mean ± SD**	52.67 ± 11.77	52.70 ± 12.06	52.66 ± 11.68	0.93
**Male, n (%)**	2632 (90)	683 (90.7)	1949 (89.6)	0.39
**Medical History, n (%)**				
**Angina/Myocardial infarction**	426 (14.5)	68 (9)	358 (16.5)	< .001
**PCI**	187 (6.4)	28 (3.7)	159 (7.3)	< .001
**CABG**	26 (0.9)	6 (0.8)	20 (0.9)	0.76
**Heart failure**	56 (1.9)	17 (2.3)	39 (1.8)	0.42
**Stroke**	81 (2.8)	19 (2.5)	62 (2.8)	0.64
**Chronic renal failure**	54 (1.8)	10 (1.3)	44 (2)	0.22
**Diabetes mellitus**	1247 (42.6)	296 (39.4)	951 (43.7)	0.04
**Hypertension**	1263 (43.1)	309 (41)	954 (44)	0.18
**Dyslipidemia**	901 (30.8)	191 (25.4)	710 (32.6)	< .001
**Current/Ex-smoking**	1532 (52.3)	400 (53.1)	1132 (52)	0.61

STEMI: ST-elevation myocardial infarction; PCI: percutaneous coronary intervention; CABG: coronary artery bypass graft surgery

The majority (almost 80%) had either no or a low level of education (illiterate/primary school education), and over half had a relatively low SES. We found a past medical history of angina or MI in 14.6%, hypertension in 43.1%, diabetes mellitus in 42.6%, and dyslipidemia in 30.8%; also, 52.3% were current/ex-smokers.

One-third of the patients had a first medical contact prior to hospital arrival (PCI or non-PCI). Of these, 48% had gone to clinics and 52% to non-PCI hospitals. Upon hospital arrival, the median heart rate was 83 beats per min, the median systolic blood pressure was 135 mmHg, and Killip classes III/IV were present in 2.9% and 3%; respectively. Median (IQR) delay times were 120 (180) min from symptom onset to first medical contact and 175 (250) min from symptom onset to ED arrival. Most patients (89.3%) arrived at the hospital in less than 12 h after symptom onset. TT was administered in 38.6% of patients, PPCI in 46% of patients, and the remaining patients did not receive either therapy, due to issues of patient eligibility for hospital admission (43.2%) or late presentation (44.6%). The median (IQR) DNT was 41 (40) min. About one-third (31%) of the patients received TT within 30 min of ED arrival, and 24.4% received emergency rescue PCI for failed reperfusion with TT. The median (IQR) DBT was 75 (57) min, and 65.5% of patients received the first balloon inflation within 90 min of ED arrival. Echocardiographic evidence of left ventricular systolic dysfunction was found in 70% of patients; among these, one-third had moderate or severe dysfunction (S1 Table).

### EMS System Features

EMS was used by 25.7% of patients with acute STEMI (Table 1). The ambulance crew qualifications were: 83.5% BLS-certified, 58.2% ACLS-certified, and 52.4% physicians. The median (IQR) durations for events between the time the patient called and the EMS response were as follows: 69.5 (120) min from symptom onset to receiving the call at the EMS center; 3 (3) min for EMS dispatch; 8 (6) min for arrival at the scene; and 18 (14) min for arrival at the ED. In the EMS group, first aid was provided by a nurse or a physician for 69.4% of patients; by a relative or a friend for 20.3% of patients; and by the Red Crescent for 10.2% of patients (i.e., only 109 patients, or 3.7% of the total acute STEMI cohort, were transferred by the Red Crescent EMS).

In the EMS group, 88% of patients received ECGs at clinics or peripheral non-PCI hospitals prior to transfer to other non-PCI or PCI-hospitals, but none had ECGs in the ambulance.

### EMS vs. Non-EMS Groups

**Fig 2:**
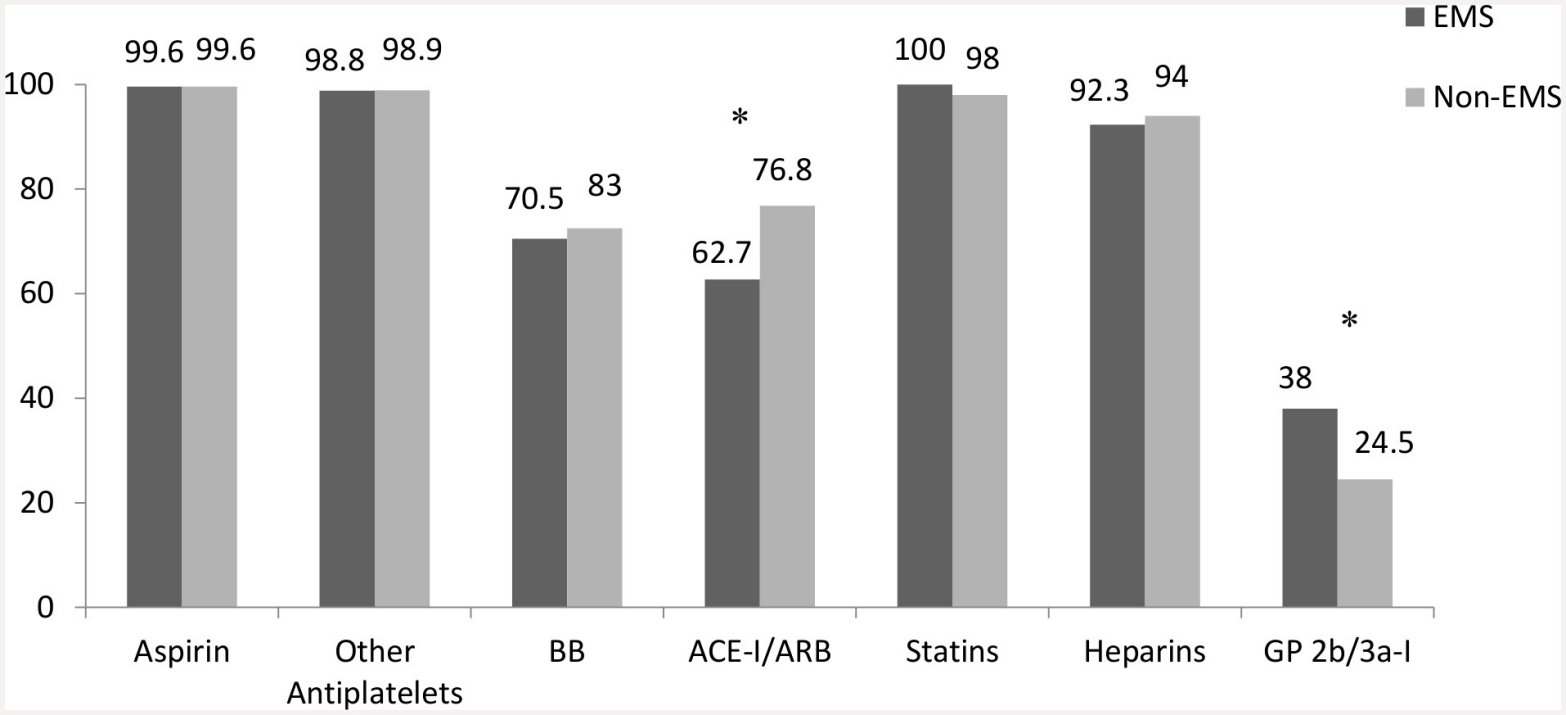
Evidence-based treatments administered in the first 24 hours of hospital admission in acute STEMI patients that arrived to the hospital by an emergency medical service (EMS) versus not (non-EMS). Other aniplatelets, clopidogrel, prasugrel, ticagrelor; BB, beta-blockers; ACE-I/ARB, angiotensin-converting enzyme inhibitors/Angiotensin-receptor blockers, Heparins, unfractionated or low-molecular weight heparin; GP 2b/3a-I, glycoprotein 2bb/3a inhibitors.

**Fig 3:**
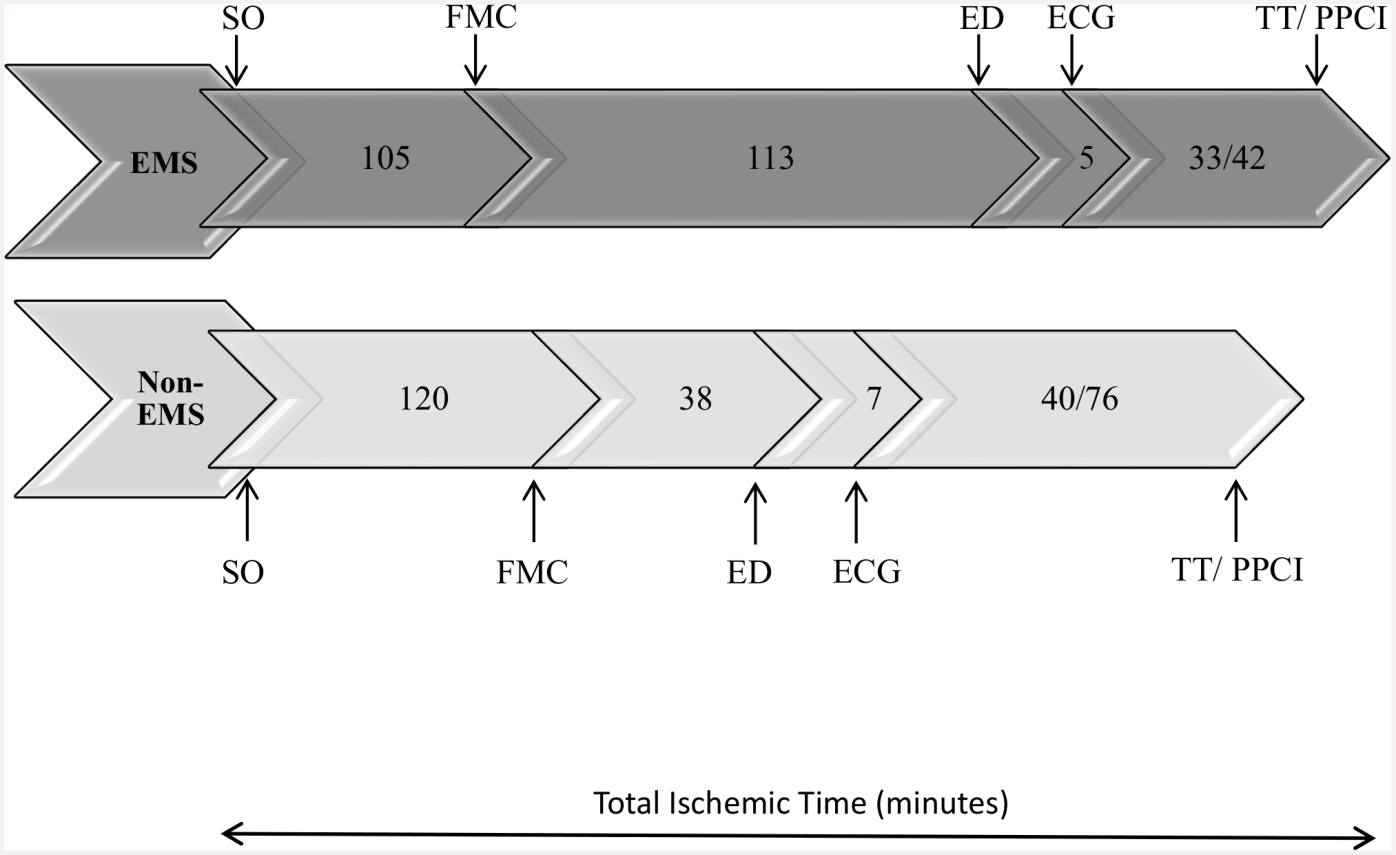
Median time-line of events from symptoms-onset to the administration of reperfusion therapies (total ischemic time) in acute STEMI patients that arrived to the hospital by an emergency medical service (EMS) versus not (non-EMS). SO, symptoms-onset, FMC, first medical contact; ED, Emergency Department arrival, ECG, electrocardiogram; TT/PPCI, thrombolytic therapy/primary percutaneous coronary intervention.

Patients in the non-EMS and EMS groups were similar in age and gender. However, compared to the non-EMS group, patients in the EMS group were less likely to have a history of diabetes mellitus (39.4% vs. 43.7%, *p* = 0.037), dyslipidemia (25.4% vs. 32.6%, *p* <0.001), or angina/MI (9% vs. 16.5%, *p* <0.001). Both the non-EMS and EMS groups received similar evidence-based treatments in the first 24 h of hospital admission. Compared to the non-EMS group, the EMS group had lower rates of angiotensin-converting enzyme inhibitors/angiotensin-receptor blocker administration (62.7% vs. 76.8%, *p* <0.001), and higher rates of glycoprotein 2b/3a-inhibitor administration (38% vs. 24.5%, *p* <0.001) (Fig 2); they were more likely to make first medical contact before arriving at the hospital (68.8% vs. 18.4%, *p* <0.001); they had longer median symptom-onset-to-ED times (218 min. vs. 158 min., *p* <0.001); they were more likely to receive PPCI (62% vs. 40.5%, *p* = 0.02); and, upon arrival to the hospital, they had shorter DNTs and DBTs (Fig 3).

The main reasons that patients did not receive either TT or PPCI were patient ineligibility for hospital admission or treatment, in the EMS group (71.7%); and late-presentation in the non-EMS group (53.3%). Among the subgroups of patients that used EMS or non-EMS and arrived at either PCI- or non-PCI hospitals, the EMS group that arrived at non-PCI hospitals had the longest median symptom-onset-to-ED time (254 min) (S2 and S3 Tables). The Red Crescent EMS rarely transferred acute STEMI patients (3.7%), had the shortest symptom-onset-to-ED time (144 min) but no significant impact on in-hospital outcomes compared to the Inter-Hospital EMS and non-EMS groups (S4 Table).

### Predictors and Outcomes

Univariate and multivariate logistic regressions were performed to identify predictors of EMS use (Table 2). We found that EMS use was mostly predicted by primary/secondary school educational levels, low/moderate SES, and “other” ethnicity. There was no significant interaction between education level and income. In contrast, a history of angina and a history of PCI predicted lower EMS use.

**Table 2:** Univariate and multivariate predictors of emergency medical service (EMS) use among patients with acute STEMI.

Predictors	Univariate Crude OR (95% CI)	*p*-value	Multivariate Adjusted OR (95% CI)	*p*-value
**Age (1-year increase)**	1.00 (0.99–1.01)	0.93		
**Gender**				
Female	0.88 (0.67–1.17)	0.39		
Male	Reference			
**Education**				
Primary School/Secondary School	1.34 (1.08–1.66)	0.01	1.83 (1.37–2.46)	< .001
Diploma/ University/ Master /PhD	1.74 (1.36–2.24)	< .001	1.42 (1.14–1.77)	0.002
Illiterate	Reference			
**Citizenship**				
Gulf	0.99 (0.83–1.17)	0.87		
Non-Gulf	Reference			
**Ethnicity**				
Arab	0.76 (0.59–0.96)	0.023	0.94 (0.71–1.23)	0.63
South Asian	0.70 (0.55–0.88)	0.003	0.73 (0.56–0.94)	0.02
Other	Reference			
**Average household monthly income**				
< $1000	1.19 (0.85–1.64)	0.308	1.64 (1.12–2.41)	0.01
$1000–$5000	1.45 (1.04–2.02)	0.031	1.62 (1.15–2.28)	0.01
> $5000	Reference			
**Type of STEMI**				
Anterior	0.87 (0.62–1.21)	0.41		
Inferior	0.78 (0.55–1.09)	0.14		
other	Reference			
**Diabetes mellitus**	0.83 (0.71–0.99)	0.04		
**Hypertension**	0.89 (0.75–1.05)	0.18		
**History of MI**	0.56 (0.39–0.81)	0.002		
**History of angina**	0.46 (0.33–0.63)	< .001	0.51 (0.36–0.71)	< .001
**History of PCI**	0.49 (0.32–0.74)	< .001	0.62 (0.40–0.96)	0.03

STEMI: ST-elevation myocardial infarction; PCI: percutaneous coronary intervention

In-hospital outcomes were similar between the two groups. The hospital stay (median [IQR]) was shorter in the EMS group (2 [3] days) than in the non-EMS group (3 [2] days; *p* = 0.008) (Table 3).

**Table 3:** In-hospital outcomes, complications, and mortality in patients with acute STEMI transported to the hospital by an emergency medical service (EMS) or alternative transportation (non-EMS).

	Total n (%)	EMS n (%)	Non-EMS n (%)	*p*-value
**Recurrent ischemia**	189 (6.5)	41 (5.6)	148 (7)	0.22
**Recurrent myocardial infarction**	43 (1.5)	7 (0.9)	36 (1.7)	0.16
**Atrial fibrillation or flutter**	63 (2.2)	15 (2)	48 (2.2)	0.76
**Heart failure**	388 (13.4)	83 (11.2)	305 (14.1)	0.05
**Cardiogenic shock**	211 (7.3)	61 (8.3)	150 (7)	0.24
**Ventricular tachycardia or fibrillation**	200 (6.9)	62 (8.4)	138 (6.4)	0.06
**Stroke**	23 (0.8)	4 (0.5)	19 (1)	0.37
**Major bleeding**	45 (1.5)	11 (1.5)	34 (1.6)	0.87
**Stent thrombosis**	20 (0.7)	4 (0.5)	16 (0.7)	0.57
**Mortality**	170 (5.8)	51 (6.8)	119 (5.5)	0.19

STEMI: ST-elevation myocardial infarction

However, crude and adjusted in-hospital mortality rates were higher in both groups that arrived at non-PCI hospitals compared to those that arrived at PCI hospitals; the highest mortality (14.7%) was observed in the EMS group that arrived at non-PCI hospitals (S2 and S3 Tables).

## Discussion

The present report of the Gulf RACE-3Ps study was the first in our region to provide results from a systematic exploration of EMS care for patients with acute STEMI. Our results confirmed our previous findings that, among patients with STEMI in our region, most were relatively young males, and two-thirds were non-Gulf citizens; moreover, these patients had a high prevalence of coronary artery disease risk factors [17]. The current report also showed that most of these patients had relatively low SES and educational levels. This finding was most likely related to the fact that large sectors of the population that live in the Arabian Gulf countries (30% in Saudi Arabia, 60% in Kuwait, 80% in UAE and Qatar) are foreign “blue-collar” workers, with limited access to health care. This observation was supported by our finding that half of the patients with acute STEMI that did not receive acute reperfusion therapies had been subject to “hospital eligibility issues” and limited access to hospital admission and treatment. This highlights the urgent need for major changes in health care policy, including adoption of universal (foreign workers included) health care insurance coverage. In particular, health insurance should cover life-saving procedures, such as reperfusion therapy for patients with acute STEMI.

We showed that the median time delay from symptom-onset-to-ED arrival was 175 min. System delay (sum of EMS and DBT delay) has not changed much over the years. In our study, the system delay was 130 min (mainly related to EMS delay), which was within the range of 60 to 177 min reported in the Stent for Life survey that was undertaken in 30 European countries [3]. In addition, we observed patient-related delays (median, 120 min), which contributed to the other half of the total ischemic time prolongation. These delays would be considered in the “high range”, compared to patient-related delays reported in other international studies. In the GRACE registry, patient-related delays were 104 to 120 min [19]; in France, delays decreased from 120 to 74 min over a 10- year period in a nation-wide registry [20]. Furthermore, among our patients with STEMI, only 30% had DNTs ≤30 min, 65% had DBTs ≤90 min, and only 25% had rescue PCIs for failed reperfusion. However, in a large CathPCI Registry in the USA, which represented patients from 2005 to 2009, the in-hospital mortality had remained unchanged, despite an increase (from 60% to 83%) in the percentage of patients with DBTs ≤ 90 min [21]. A re-analysis of the same registry for patients between 2005 and 2011 showed that patient-specific DBTs were associated with lower mortality over time, but there were trends for increased mortality risk at the population level [22]. Thus, major emphasis should be placed on prompt initiation of acute reperfusion therapies upon first medical contact as well as improving the total ischemic time. It is essential to launch public educational campaigns to raise public awareness of acute coronary syndromes symptoms and the importance of early arrival to the nearest hospital, particularly hospitals that offer 24/7 PPCI-capability.

Around one in four patients with acute STEMI arrived at the ED by EMS. This rate did not improve over the rate found in our previous report. A more disturbing finding was the fact that only 10.2% of EMS use was via the Red Crescent (or 3.7% of the overall acute STEMI cohort). This is an extremely low utilization rate; almost all patients that used EMS were transported in ambulances that operated under the authority of clinics and/or non-PCI hospitals. Thus, it was not surprising to find that the EMS response times were quite rapid, because these were clinic/hospital-related ambulances that transferred patients to other hospitals after patients had arrived at the hospital in their own cars. In contrast, ambulances operated by the Red Crescent had transferred patients directly from the scene to the hospital. In a Swedish multicenter study, only 51% reported that the first medical contact was with EMS; 14% went directly to the ED; and the remaining patients chose to discuss their symptoms first by either going to a clinic or consulting a public health care advisory service [23]. In a recent registry of 14,518 patients with STEMI in the USA, only 25% used EMS, and more than one third of those transferred for PPCIs failed to achieve a first door-to-device time ≤120 min, despite estimated transfer times of ˂60 min [24]. This study also clearly demonstrated that high-volume STEMI PCI centers were 20% more likely than low-volume centers to achieve timely acute reperfusion.

A significant proportion of the ambulance paramedics in our study lacked BLS and ACLS certifications. Most ECGs were performed in the clinics or non-PCI hospitals, rather than in the ambulance. It has been shown that, when pre-hospital ECGs were transmitted to hospitals by the EMS, the total ischemic time could be reduced and false-positive activations of the PCI hospital could be avoided [25–28]. State-of-the-art tertiary care hospitals were established in our region over the last few decades. The new health care system priorities should be to improve primary prevention of cardiovascular diseases and establish an efficient, robust EMS infrastructure under the authority of a single national health care provider.

Patients in the EMS group were less likely than those in the non-EMS group to have coronary artery disease risk factors or a history of ischemic heart disease. Therefore, the majority of patients that used EMS were most likely uncertain of their diagnosis when they became symptomatic. Consequently, they were self-transported, first to a clinic or a peripheral non-PCI hospital; then, subsequently, they were transferred by ambulance to another, larger non-PCI hospital or to a PCI hospital. Therefore, unlike patients in western countries with well-developed EMS systems and organized STEMI networks, our patients that used EMS were mostly in the Inter-Hospital EMS group rather than in the Red Crescent EMS group, and subsequently had to go through “several stops” prior to being diagnosed and transferred for acute reperfusion; thus, these patients experienced longer total ischemic times than patients that did not use EMS. The delay in arrival by EMS might have off-set a potential benefit in clinical outcomes gained with shorter DNTs and DBTs. In fact, in the EMS group, the mortality rate was much higher for those that initially went to non-PCI hospitals than for those that initially went to PCI hospitals (14.7% vs. 5.2%). However, this difference in mortality was most likely related to a combination of factors, including the disease severity in the patient population, the pre-hospital delay times, and the relatively lower standards of care in the non-PCI hospitals. Furthermore, low to moderate SES and “other” ethnicity were predictors of EMS use; thus, many patients with acute STEMI, particularly foreign workers, might have tried to access hospital care by arriving in an ambulance, because otherwise, they would be declined admission to the ED; in contrast, Gulf citizens and/or those with a history of ischemic heart disease relied on rapid self-transport to the nearest hospital, because they were unlikely to face eligibility issues regarding their hospital admission or management.

### Limitations

Our study had a few limitations. As with most other registries, hospital enrolment was voluntary; thus, our observations may not be representative of clinical practice in all hospitals in the region or in a particular country. It is likely that the actual lack of knowledge about care was greater in our region than that reported here, considering that many of the centers included in our study were highly advanced PCI hospitals. However, we strived to include a reasonable mix of PCI- and non-PCI hospitals in the study as a whole, and we enrolled consecutive patients with acute STEMI, including transfers from other clinics or hospitals. The sample of cardiac centers included in our study represented approximately half of the hospitals currently operating in large Arabian Gulf countries and all cardiac centers in the relatively smaller countries. In addition, the hospitals included in this study were from different health care sectors and geographic locations; thus, this combination was expected to provide a reasonable representation of the STEMI care throughout our region (S1 Fig). Also, observational studies, like the present study, are typically confounded by unmeasured variables, such as physician- or patient-related choices of a particular therapeutic strategy. Nevertheless, the present study included all the standard variables used in our daily clinical practice, in addition to other, non-clinical variables, such as patient ethnicity, education, and SES levels. Lastly, our study results could not be generalized to all patients with acute coronary syndromes since it included only patients with acute STEMI.

### Conclusions

This study revealed that most patients with acute STEMI in the Arabian Gulf region did not use EMS. In this region, the top health care system priorities should be to improve Red Crescent infrastructure, establish integrated STEMI networks, and launch public education campaigns.

## Supporting Information

S1 Fig Regional map of the 22 PCI centers enrolled in the study. (TIF)

S1 Table Detailed time-line of events from symptoms-onset to the administration of reperfusion therapies, and procedures in acute STEMI patients that arrived to the hospital by an emergency medical service (EMS) versus not (Non-EMS). (DOCX)

S2 Table Demographics, clinical presentation, educational and socioeconomic characteristics, management, and in-hospital outcomes of patients with acute STEMI that arrived to PCI- versus non-PCI hospitals by an emergency medical service (EMS) versus not (Non-EMS). (DOCX)

S3 Table Adjusted in-hospital outcomes of patients with acute STEMI that arrived to PCI- versus non-PCI hospitals. (DOCX)

S4 Table Demographics, clinical presentation, management, and in-hospital outcomes of patients with acute STEMI transported to the hospital by the Red Crescent Emergency Medical Service (EMS) versus Inter-Hospital EMS versus non-EMS. (DOCX)

S1 Text List of *Gulf RACE-3Ps* Co-Investigators and Research Assistants (other than the co-authors). (DOCX)
