# Supplementary figures and images for: Patient and System-Related Delays of Emergency Medical Services Use in Acute ST-Elevation Myocardial Infarction: Results from the Third Gulf Registry of Acute Coronary Events (Gulf RACE-3Ps)
Source: PLoS One. 2016 Jan 25;11(1):e0147385. doi: 10.1371/journal.pone.0147385 (PMC4726591; doi:10.1371/journal.pone.0147385)

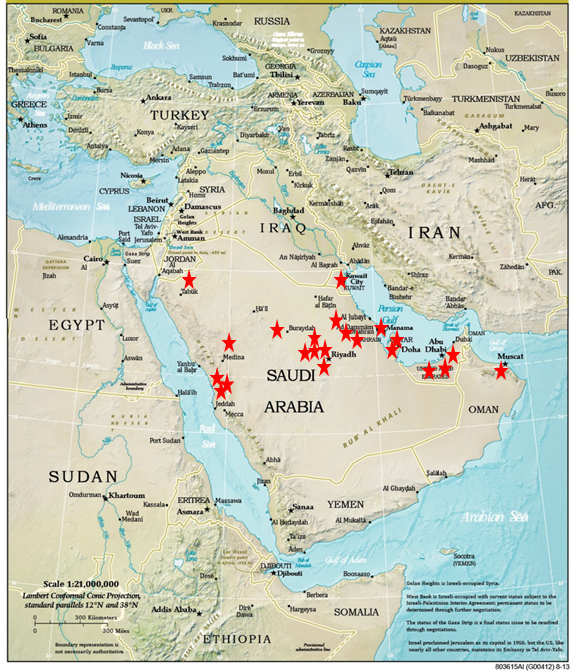

Supplement: S1 Fig — (TIF) [file pone.0147385.s001.tif]
