# Supplementary material for: Patient and System-Related Delays of Emergency Medical Services Use in Acute ST-Elevation Myocardial Infarction: Results from the Third Gulf Registry of Acute Coronary Events (Gulf RACE-3Ps)
Source: PLoS One. 2016 Jan 25;11(1):e0147385. doi: 10.1371/journal.pone.0147385 (PMC4726591; doi:10.1371/journal.pone.0147385)
Supplement: S1 Table — (DOCX) [file pone.0147385.s002.docx]

**S1 Table** Detailed time-line of events from symptoms-onset to the administration of reperfusion therapies, and procedures in acute STEMI patients that arrived to the hospital by an emergency medical service (EMS) versus not (Non-EMS)

|  | **Total** | **EMS** | **Non-EMS** | **P-value** |
| --- | --- | --- | --- | --- |
| Symptoms to first medical contact time, median (IQR), min | 120 (180) | 105 (150) | 120 (226) | 0.18 |
| Symptoms to ED time, median (IQR), min | 175 (250) | 218.0 ( 256 ) | 158 (241) | <.001 |
| Symptoms to ED time ≤12hrs, n (%) | 2550 (89.3) | 632 (88.6) | 1918 (89.5) | 0.50 |
| ED to diagnostic ECG, median (IQR), min | 7 (7) | 5 (8) | 7 (8) | <.001 |
| ED to diagnostic ECG ≤10 Minute, n (%) | 2101 (73.5) | 563 (78.8) | 1538 (71.8) | <.001 |
| **Thrombolytic therapy, n (%)** | 1117 (38.6) | 149 (20.2) | 968 (45) | <.001 |
| DNT, median (IQR), min | 41 (40) | 38 (35) | 42 (41) | 0.04 |
| DNT≤ 30 Minute, n (%) | 344 (30.8) | 53 (35.6) | 291 (30.1) | 0.17 |
| Clinical signs of reperfusion, n (%) | 911 (81.6) | 124 (83.2) | 787 (81.3) | 0.57 |
| Rescue PCI for failed clinical reperfusion, n (%) | 54 (24.4) | 6 (22.2) | 48 (24.7) | 0.77 |
| **Primary PCI, n (%)** | 1347 (46) | 466 (62) | 881 (40.5) | 0.02 |
| DBT, median (IQR), min | 75 (57) | 47 (51) | 83 (53) | <.001 |
| DBT ≤ 90 Minutes, n (%) | 865 (65.5) | 366 (82.2) | 499 (57) | <.001 |
| **Why not Thrombolytic therapy or Primary PCI?** | | | | |
| Late-presentation, n (%) | 189 (44.6) | 27 (22.5) | 162 (53.3) | <.001 |
| Missed, n (%) | 25 (6) | 3 (2.5) | 22 (7.2) |  |
| Contraindication, n (%) | 27 (6.4) | 4 (3.3) | 23 (7.6) |  |
| Other (e.g: patients’ eligibility issues), n (%) | 183 (43.2) | 86 (71.7) | 97 (32) |  |
| **CABG, n (%)** | 59 (2) | 10 (1.4) | 49 (2.3) | 0.13 |
| **Echocardiography, n (%)** | 2790 (96.3) | 717 (97.2) | 2073 (96) | 0.17 |
| Normal LV systolic function (EF >50%), n (%) | 849 (30.5) | 194 (27.2) | 655 (31.7) | 0.10 |
| Mild LV systolic dysfunction (EF 40-50%), n (%) | 1108 (39.8) | 296 (41.5) | 812 (39.3) |  |
| Moderate LV systolic dysfunction (EF 30-40%), n (%) | 592 (21.3) | 155 (21.7) | 437 (21.1) |  |
| Severe LV systolic dysfunction (EF <30%), n (%) | 233 (8.4) | 69 (9.7) | 164 (8) |  |

ED, emergency department; ECG, electrocardiogram; DNT, door-to-needle time; DBT, door-to-balloon time; PCI, percutaneous coronary intervention; Coronary artery bypass graft, CABG; LV, left ventricular
