# Supplementary material for: Patient and System-Related Delays of Emergency Medical Services Use in Acute ST-Elevation Myocardial Infarction: Results from the Third Gulf Registry of Acute Coronary Events (Gulf RACE-3Ps)
Source: PLoS One. 2016 Jan 25;11(1):e0147385. doi: 10.1371/journal.pone.0147385 (PMC4726591; doi:10.1371/journal.pone.0147385)
Supplement: S2 Table — (DOCX) [file pone.0147385.s003.docx]

**S2 Table** Demographics, clinical presentation, educational and socioeconomic characteristics, management, and in-hospital outcomes of patients with acute STEMI that arrived to PCI- versus non-PCI hospitals by an emergency medical service (EMS) versus not (Non-EMS)

|  | **PCI Hospitals**  **N=1931(65.95%)** | | | | **Non-PCI Hospitals**  **N=997(34.05%)** | | | |
| --- | --- | --- | --- | --- | --- | --- | --- | --- |
|  | **Total**  **n=1931** | **EMS n=631(32.68%)** | **Non-EMS**  **n=1300 (67.32%)** | **P-value** | **Total**  **n=997** | **EMS**  **n=122 (12.24%)** | **Non-EMS n=875 (87.76%)** | **P-**  **value** |
| Age | 52.43 ± 11.40 | 52.51 ± 11.85 | 52.39 ± 11.18 | 0.82 | 53.13 ± 12.46 | 53.65 ± 13.08 | 53.06 ± 12.38 | 0.62 |
| Male | 1744(90.32%) | 574 (90.97%) | 1170 (90%) | 0.5 | 888 (89.1%) | 109 (89.34%) | 779 (89.03%) | 0.92 |
| Citizenship* |  |  |  |  |  |  |  |  |
| Gulf | 728 (37.7%) | 227 (31.18%) | 501 (68.82%) | 0.28 | 395 (39.62%) | 60 (15.19%) | 335 (84.81%) | 0.02 |
| Non-Gulf | 1203 (62.3%) | 404 (33.58%) | 799 (66.42%) |  | 602 (60.38%) | 62 (10.3%) | 540 (89.7%) |  |
| Education* |  |  |  |  |  |  |  |  |
| Illiterate | 383 (19.83%) | 115 (30.03%) | 268 (69.97%) | 0.23 | 334 (33.5%) | 33 (9.88%) | 301 (90.12%) | 0.14 |
| Primary School/Secondary School | 1033 (53.5%) | 334 (32.33%) | 699 (67.67%) |  | 563 (56.47%) | 79 (14.03%) | 484 (85.97%) |  |
| Diploma/university/Master/PhD | 515 (26.67%) | 182 (35.34%) | 333 (64.66%) |  | 100 (10.03%) | 10 (10%) | 90 (90%) |  |
| Average Household Monthly Income (US $)* |  |  |  |  |  |  |  |  |
| < 1000$ | 1051(54.43%) | 332 (31.59%) | 719 (68.41%) | 0.01 | 604 (60.58%) | 75 (12.42%) | 529 (87.58%) | 0.10 |
| 1000 - 5000 $ | 684 (35.42%) | 248 (36.26%) | 436 (63.74%) |  | 348 (34.90%) | 46 (13.22%) | 302 (86.78%) |  |
| > 5000 $ | 196 (10.15%) | 51 (26.02%) | 145 (73.98%) |  | 45 (4.51%) | 1 (2.22%) | 44 (97.78%) |  |
| Type of STEMI* |  |  |  |  |  |  |  |  |
| Anterior | 1045(54.12%) | 353 (33.78%) | 692 (66.22%) | 0.08 | 528 (52.96%) | 62 (11.74%) | 466 (88.26%) | 0.74 |
| Inferior | 760 (39.36%) | 229 (30.13%) | 531 (69.87%) |  | 403 (40.42%) | 53 (13.15%) | 350 (86.85%) |  |
| other | 126 (6.53%) | 49 (38.89%) | 77 (61.11%) |  | 66 (6.62%) | 7 (10.61%) | 59 (89.39%) |  |
| Diabetes mellitus | 832 (43.11%) | 253 (40.16%) | 579 (44.54%) | 0.07 | 415 (41.62%) | 43 (35.25%) | 372 (42.51%) | 0.13 |
| Hypertension | 833 (43.14%) | 265 (42.00%) | 568 (43.69%) | 0.48 | 430 (43.13%) | 44 (36.07%) | 386 (44.11%) | 0.09 |
| Current/Ex smoking | 1032(53.44%) | 336 (53.25%) | 696 (53.54%) | 0.91 | 500 (50.15%) | 64 (52.46%) | 436 (49.83%) | 0.59 |
| Dyslipidemia | 561 (29.05%) | 158 (25.04%) | 403 (31.00%) | 0.01 | 340 (34.10%) | 33 (27.05%) | 307 (35.09%) | 0.08 |
| History of myocardial infarction /Angina | 273 (14.14%) | 51 (8.08%) | 222 (17.08%) | <.001 | 153 (15.35%) | 17 (13.93%) | 136 (15.54%) | 0.64 |
| History of PCI | 138 (7.15%) | 25 (3.96%) | 113 (8.69%) | <.001 | 49 (4.91%) | 3 (2.46%) | 46 (5.26%) | 0.18 |
| History of stroke | 50 (2.59%) | 11 (1.74%) | 39 (3%) | 0.1 | 31 (3.11%) | 8 (6.56%) | 23 (2.63%) | 0.02 |
| Symptoms onset to first medical contact, median (IQR), min | 117.0 (160) | 108.5 (150) | 120.0 (230) | 0.8 | 120.0 (202.5) | 90.00 (160) | 120.0 (240) | 0.11 |
| Symptoms onset to ED time, Median(IQR), min | 155.0 (241) | 210.0 (230) | 130.0 (227) | <.001 | 200.0 (262) | 254.0 (366) | 192.0 (250) | 0.01 |
| Symptoms onset to ED time ≤12hrs | 1695(90.84%) | 529 (89.51%) | 1166 (91.45%) | 0.18 | 855 (86.45%) | 103 (84.43%) | 752 (86.74%) | 0.49 |
| ED to Diagnostic ECG, Median(IQR), min | 6.00 (8) | 5.00 (8) | 6.00 (8) | <.001 | 8.00 (6) | 6.00 (5) | 8.00 (7) | 0.05 |
| ED to Diagnostic ECG ≤10 Minute | 1389 (74.36%) | 468 (79.05%) | 921 (72.18%) | 0.002 | 712 (71.99%) | 95 (77.87%) | 617 (71.16%) | 0.12 |
| Thrombolytic therapy given | 312 (16.36%) | 47 (7.63%) | 265 (20.53%) | <.001 | 805 (81.40%) | 102 (83.61%) | 703 (81.08%) | 0.50 |
| DNT, Median(IQR) | 40 (44) | 33.00 (27) | 42 (42) | 0.06 | 42.00 (41) | 40.5 (40) | 42 (42) | 0.28 |
| DNT time ≤ 30 Minute | 111 (35.58%) | 23 (48.94%) | 88 (33.21%) | 0.04 | 233 (28.94%) | 30 (29.41%) | 203 (28.88%) | 0.91 |
| Primary PCI done (if thrombolytic therapy not given) | 1324(83.43%) | 466 (82.33%) | 858 (84.04%) | 0.38 | ------ | ------ | ------ | ------ |
| DBT, Median(IQR), min | 74 (57) | 47.00 (51) | 82.5 (53) | <.001 | 98 (77) |  | 98 (77) |  |
| DBT ≤ 90 Minutes | 856 (66%) | 366 (82.25%) | 490 (57.51%) | <.001 | 9 (39.13%) |  | 9 (39.13%) |  |
| Recurrent ischemia | 82 (4.30%) | 19 (3.08%) | 63 (4.88%) | 0.07 | 107 (10.82%) | 22 (18.03%) | 85 (9.80%) | 0.01 |
| Recurrent myocardial infarction | 21 (1.10%) | 6 (0.97%) | 15 (1.16%) | 0.71 | 22 (2.22%) | 1 (0.82%) | 21 (2.42%) | 0.26 |
| Atrial Fibrillation/Flutter | 39 (2.05%) | 9 (1.46%) | 30 (2.32%) | 0.21 | 24 (2.43%) | 6 (4.92%) | 18 (2.08%) | 0.06 |
| Heart Failure | 209 (10.96%) | 65 (10.55%) | 144 (11.15%) | 0.69 | 179 (18.10%) | 18 (14.75%) | 161 (18.57%) | 0.31 |
| Cardiogenic Shock | 127 (6.66%) | 46 (7.47%) | 81 (6.27%) | 0.33 | 84 (8.49%) | 15 (12.30%) | 69 (7.96%) | 0.11 |
| VT/VF arrest | 116 (6.08%) | 44 (7.14%) | 72 (5.58%) | 0.18 | 84 (8.49%) | 18 (14.75%) | 66 (7.61%) | 0.01 |
| Stroke | 13 (0.68%) | 3 (0.49%) | 10 (0.77%) | 0.48 | 10 (1.01%) | 1 (0.82%) | 9 (1.04%) | 0.82 |
| Major bleeding | 28 (1.47%) | 10 (1.62%) | 18 (1.39%) | 0.7 | 17 (1.72%) | 1 (0.82%) | 16 (1.85%) | 0.41 |
| Stent thrombosis | 15 (0.79%) | 4 (0.65%) | 11 (0.85%) | 0.64 | 5 (0.51%) | 0 (0.00%) | 5 (0.58%) | 0.4 |
| CABG | 58 (3.04%) | 10 (1.62%) | 48 (3.72%) | 0.01 | ----- | ----- | ----- | ----- |
| In-hospital mortality | 86 (4.45%) | 33 (5.23%) | 53 (4.08%) | 0.25 | 84 (8.43%) | 18 (14.75%) | 66 (7.54%) | 0.01 |
| Length of stay, median (days) | 3 (2) | 2 (3) | 3 (2) | 0.004 | 3 (2) | 3 (3) | 3 (2) | 0.05 |

* Row percentages

PCI: percutaneous coronary intervention, CABG: coronary artery bypass graft surgery
