# Supplementary material for: Patient and System-Related Delays of Emergency Medical Services Use in Acute ST-Elevation Myocardial Infarction: Results from the Third Gulf Registry of Acute Coronary Events (Gulf RACE-3Ps)
Source: PLoS One. 2016 Jan 25;11(1):e0147385. doi: 10.1371/journal.pone.0147385 (PMC4726591; doi:10.1371/journal.pone.0147385)
Supplement: S3 Table — (DOCX) [file pone.0147385.s004.docx]

**S3 Table** Adjusted in-hospital outcomes of patients with acute STEMI that arrived to PCI- versus non-PCI hospitals

| **Outcomes** | **Level** | **Crude OR(95% CI)** | **P-value** | **Adjusted OR (95% CI)** | **P-value** |
| --- | --- | --- | --- | --- | --- |
| Mortality | Non-PCI Hospitals | 1.97(1.446,2.69) | <.001 | 2.18(1.397,3.41) | <.001 |
| Heart Failure | Non-PCI Hospitals | 1.80(1.446,2.23) | <.001 | 1.76(1.286,2.41) | <.001 |
| VT/VF arrest | Non-PCI Hospitals | 1.43(1.07,1.92) | 0.016 | 1.82(1.202,2.76) | 0.005 |
| Recurrent MI | Non-PCI Hospitals | 2.04(1.118,3.73) | 0.020 | 1.44(0.647,3.21) | 0.371 |

Adjustments for: Education, monthly income, dyslipidemia, history of PCI, thrombolytic therapy, symptoms onset to emergency department time, Medications in the first 24hrs of hospital admission: Clopidogrel, Ticagrelor, Beta-Blockers, ACE-I or ARB
