# Supplementary material for: Patient and System-Related Delays of Emergency Medical Services Use in Acute ST-Elevation Myocardial Infarction: Results from the Third Gulf Registry of Acute Coronary Events (Gulf RACE-3Ps)
Source: PLoS One. 2016 Jan 25;11(1):e0147385. doi: 10.1371/journal.pone.0147385 (PMC4726591; doi:10.1371/journal.pone.0147385)
Supplement: S4 Table — (DOCX) [file pone.0147385.s005.docx]

**S4 Table** Demographics, clinical presentation, management, and in-hospital outcomes of patients with acute STEMI transported to the hospital

by the Red Crescent Emergency Medical Service (EMS) versus Inter-Hospital EMS versus non-EMS

|  | **Total**  **n= 2928** | **Red Crescent EMS**  **n= 109 (3.7%)** | **Inter-Hospital EMS**  **n= 644**  **(22%)** | **Non-EMS**  **n= 2175 (74.3%)** | **P-value** |
| --- | --- | --- | --- | --- | --- |
| **Row Percentages** | | | | | |
| **Citizenship** |  |  |  |  |  |
| Gulf | 1123 (38.35%) | 19 (1.69%) | 268 (23.86%) | 836 (74.44%) | <.001 |
| Non-Gulf | 1805 (61.65%) | 90 (4.99%) | 376 (20.83%) | 1339 (74.18%) |  |
| **Education** |  |  |  |  |  |
| Illiterate | 717 (24.49%) | 7 (0.98%) | 141 (19.67%) | 569 (79.36%) | <.001 |
| Primary School/Secondary School | 1596 (54.51%) | 63 (3.95%) | 350 (21.93%) | 1183 (74.12%) |  |
| Diploma/university/Master/PhD | 615 (21.00%) | 39 (6.34%) | 153 (24.88%) | 423 (68.78%) |  |
| **Average Household Monthly Income** |  |  |  |  |  |
| < 1000$ | 1655 (56.52%) | 67 (4.05%) | 340 (20.54%) | 1248 (75.41%) | 0.02 |
| 1000 - 5000 $ | 1032 (35.25%) | 33 (3.2%) | 261 (25.29%) | 738 (71.51%) |  |
| > 5000 $ | 241 (8.23%) | 9 (3.73%) | 43 (17.84%) | 189 (78.42%) |  |
| **Type of STEMI** |  |  |  |  |  |
| Anterior | 1573 (53.72%) | 51 (3.24%) | 364 (23.14%) | 1158 (73.62%) | <.001 |
| Inferior | 1163 (39.72%) | 39 (3.35%) | 243 (20.89%) | 881 (75.75%) |  |
| other | 192 (6.56%) | 19 (9.9%) | 37 (19.27%) | 136 (70.83%) |  |
| **Column Percentages** | | | | | |
| Age | 52.67 ± 11.77 | 50.24 ± 11.18 | 53.11 ± 12.16 | 52.66 ± 11.68 | 0.06 |
| Male | 2632 (89.89%) | 102 (93.58%) | 581 (90.22%) | 1949 (89.61%) | 0.39 |
| DM | 1247 (42.60%) | 45 (41.28%) | 251 (39.04%) | 951 (43.72%) | 0.10 |
| HTN | 1263 (43.14%) | 35 (32.11%) | 274 (42.55%) | 954 (43.86%) | 0.05 |
| Current/Ex smoking | 1532 (52.32%) | 71 (65.14%) | 329 (51.09%) | 1132 (52.05%) | 0.02 |
| Hypercholesterolemia | 901 (30.77%) | 23 (21.10%) | 168 (26.09%) | 710 (32.64%) | <.001 |
| History MI/Angina | 426 (14.55%) | 9 (8.26%) | 59 (9.16%) | 358 (16.46%) | <.001 |
| History of PCI | 187 (6.39%) | 4 (3.67%) | 24 (3.73%) | 159 (7.31%) | 0.00 |
| History of stroke | 81 (2.77%) | 3 (2.75%) | 16 (2.48%) | 62 (2.85%) | 0.88 |
| Symptom onset to first medical contact time, Median (IQR) | 120.0 ( 180.0 ) | 95.00 ( 67.00 ) | 105.0 ( 150.0 ) | 120.0 ( 226.0 ) | 0.39 |
| Symptom to ED time, Median(IQR) | 175.0 ( 247.0 ) | 144.0 ( 191.0 ) | 230.0 ( 277.0 ) | 158.0 ( 241.0 ) | <.001 |
| Symptom to ED time ≤12hrs | 2550 (89.32%) | 93 (93.00%) | 539 (87.93%) | 1918 (89.54%) | 0.25 |
| ED to Diagnostic ECG, Median(IQR) | 7.00 ( 7.00 ) | 2.00 ( 3.00 ) | 6.00 ( 7.00 ) | 7.00 ( 8.00 ) | <.001 |
| ED to Diagnostic ECG ≤10 Minute | 2101 (73.54%) | 90 (89.11%) | 473 (77.16%) | 1538 (71.77%) | <.001 |
| Thrombolytic therapy given | 1117 (38.57%) | 10 (9.26%) | 139 (22.06%) | 968 (44.86%) | <.001 |
| ED to given(DNT) time, Median(IQR) | 41.00 ( 40.00 ) | 34.50 ( 46.00 ) | 38.00 ( 35.00 ) | 42.00 ( 41.00 ) | 0.12 |
| ED to given(DNT) time ≤ 30 Minute | 344 (30.80%) | 4 (40.00%) | 49 (35.25%) | 291 (30.06%) | 0.38 |
| Clinical Signs of Reperfusion | 911 (81.56%) | 7 (70.00%) | 117 (84.17%) | 787 (81.30%) | 0.46 |
| Rescue PCI | 54 (24.43%) | 1 (25.00%) | 5 (21.74%) | 48 (24.74%) | 0.95 |
| Primary PC done | 1347 (76.06%) | 86 (88.66%) | 380 (77.71%) | 881 (74.35%) | 0.00 |
| ED to Device (DBT) time, Median(IQR) | 75.00 ( 57.00 ) | 52.00 ( 44.50 ) | 46.00 ( 54.00 ) | 83.00 ( 53.50 ) | <.001 |
| ED to Device (DBT) time ≤ 90 Minutes | 865 (65.48%) | 67 (83.75%) | 299 (81.92%) | 499 (56.96%) | <.001 |
| **In-hospital complications** |  |  |  |  |  |
| Recurrent ischemia | 189 (6.53%) | 3 (2.78%) | 38 (6.03%) | 148 (6.86%) | 0.21 |
| Recurrent MI | 43 (1.48%) | 0 (0) | 7 (1.11%) | 36 (1.67%) | 0.26 |
| Atrial Fibrillation Flutter | 63 (2.18%) | 1 (0.93%) | 14 (2.22%) | 48 (2.22%) | 0.66 |
| Heart Failure | 388 (13.40%) | 18 (16.67%) | 65 (10.32%) | 305 (14.13%) | 0.03 |
| Cardiogenic Shock | 211 (7.29%) | 9 (8.33%) | 52 (8.25%) | 150 (6.95%) | 0.50 |
| VT/VF arrest | 200 (6.91%) | 13 (12.04%) | 49 (7.78%) | 138 (6.39%) | 0.05 |
| Stroke | 23 (0.79%) | 1 (0.93%) | 3 (0.48%) | 19 (0.88%) | 0.60 |
| Major bleeding | 45 (1.55%) | 2 (1.85%) | 9 (1.43%) | 34 (1.58%) | 0.94 |
| Stent thrombosis | 20 (0.69%) | 0 (0.00%) | 4 (0.63%) | 16 (0.74%) | 0.65 |
| Mortality | 170 (5.81%) | 7 (6.42%) | 44 (6.83%) | 119 (5.47%) | 0.41 |
