# Supplementary material for: Patient and System-Related Delays of Emergency Medical Services Use in Acute ST-Elevation Myocardial Infarction: Results from the Third Gulf Registry of Acute Coronary Events (Gulf RACE-3Ps)
Source: PLoS One. 2016 Jan 25;11(1):e0147385. doi: 10.1371/journal.pone.0147385 (PMC4726591; doi:10.1371/journal.pone.0147385)
Supplement: S1 Text — (DOCX) [file pone.0147385.s006.docx]

**S1 Text** List of *Gulf RACE-3Ps* Co-Investigators and Research Assistants (other than the co-authors)

**Saudi Arabia:** Khalid Al Faraidy, Prachuap Denchu, Mubarak Aldosari, Omar Alnobani, Ahmad Hayajneh, Shukri Al-Saif, Rana Almuhawes, Hind Alkammar, Layla Al-Houti, Myra Luz Guiles, Saleh Alghamdi, Abbas Rababh, Abdelmaksoud Elganady, Rino Hipolito, Layth Mimish, Issam Altnji, Fawaz Al Mutairi, Shatha Gharawi, Gamal Hussein, Hassan Darwish. **Oman:** Mamatha Punji, Raja Rao, Prit Pal Singh, Aouf Al azzawi, Ananth Narayan, Abraham k Abraham, Mohammed khadir, Hisham Soliman, Osama Al Kadi, Mourad, Abeer metwally. **United Arab Emirates:** Asood Ghori, Afzalhussein Yusufali, Nooshin Bazargani, Arif Al Mulla, Rajeev Gupta, Amrish Agrawal, Adel Abdullah Wassef. **Qatar:** Ruby Delos Santos, Mohamed Gomaa Gad, Emad Ahmed Hassan, Fahad Al Kindi, Ali Eshtiwi Siaidawi, Omar Hassan, Farookh Haider, Imtiaz Salim, Pravesh Jaguri, Narinder Kumar, Amar Salaam. **Bahrain:** Fawaz Khalil Bardooli, Mujahed Abdulsattar Ibrahim, Heba Omar Abduljawad, Alya Salman Al Doseri. **Kuwait:** Ahmad Said Taha, Wael Sayed Abdelmotelab, Asem Abdallah Abdaleem Hemeda, Ahmad Salem, Zubair Mohamed.
